# T cell–derived exosomes induced macrophage inflammatory protein‐1α/β drive the trafficking of CD8^+^ T cells in oral lichen planus

**DOI:** 10.1111/jcmm.16020

**Published:** 2020-10-27

**Authors:** Jing‐Ya Yang, Jing Zhang, Rui Lu, Ya‐Qin Tan, Ge‐Fei Du, Gang Zhou

**Affiliations:** ^1^ The State Key Laboratory Breeding Base of Basic Science of Stomatology (Hubei‐MOST) and Key Laboratory of Oral Biomedicine Ministry of Education School and Hospital of Stomatology Wuhan University Wuhan China; ^2^ Department of Oral Medicine School and Hospital of Stomatology Wuhan University Wuhan China

**Keywords:** exosome, macrophage inflammatory protein‐1α/β, migration, oral lichen planus, T cell

## Abstract

Oral lichen planus (OLP) is a T cell–mediated chronic inflammatory disease with uncertain aetiology. Exosomes are nanosized particles with biological capacities. Here, we aimed to study the effects of T cell–derived exosomes (T‐exos) on the pathogenesis of OLP and its mechanism. T‐exos were incubated with Jurkat cells for 48 hours, and 26 cytokines in the supernatant were measured by luminex assay. The expression of macrophage inflammatory protein (MIP)‐1α/β was detected using immunohistochemistry and ELISA; that of CCR1/3/5 on peripheral T cells was determined by flow cytometry. Transwell assay was performed to investigate the chemotactic effect of MIP‐1α/β, and cells in the lower chambers were examinated by flow cytometry. As a result, OLP T‐exos elevated the production of MIP‐1α/β, which were highly expressed in OLP tissues and plasma. CCR1/5 were markedly expressed on OLP peripheral T cells, and the majority of CCR1/5^+^ T cells were CD8^+^ T cells. Besides, MIP‐1α/β promoted the migration of OLP mononuclear cells, while inhibiting CCR1/5 significantly decreased the trafficking of mononuclear cells, especially that of CD8^+^ T cells. Conclusively, OLP T‐exos‐induced MIP‐1α/β may drive the trafficking of CD8^+^ T cells after binding with CCR1/5 in OLP, contributing to the development of OLP.

## 1 INTRODUCTION

Oral lichen planus (OLP) is a chronic inflammatory disease with autoimmune features, involving mainly the oral mucosa with unknown aetiology.^1^ It affects 0.5%‐3% of the worldwide population with female predilection and has been recognized as an oral potentially malignant disorder by WHO.^2^ Histopathologically, OLP is characterized by a dense subepithelial infiltrate of T lymphocytes, increased amounts of intra‐epithelial T lymphocytes, basal keratinocytes degeneration and the basement membrane disruption.^3^ Although the pathogenesis remain unknown, evidence suggests that the progression of OLP depends on a T cell–dominated antigen‐specific mechanism.^1^ In this process, cytokines play a vital role in determining the characterization and function of immune responses.^4^ Chemokines released by activated T cells and keratinocytes are able to elicit the migration of CD8^+^ T cells into the epithelium, and the followed cytotoxic CD8^+^ T cell response mediates the apoptosis of keratinocytes.^3^, ^5^ Chemokines mediated CD8^+^ T‐cell recruitment and cytokines orchestrated inflammatory response are believed to be responsible for the sustained inflammation in OLP lesions.^4^, ^5^, ^6^

Multivesicular bodies (MVBs) are unique organelles in the endocytic pathway that contain intraluminal vesicles (ILVs) in their lumen.^7^ The formation of MVBs and ILVs is certainly complex, being driven by the endosomal sorting complex required for transport (ESCRT) composed of approximately 30 proteins that assemble into 4 complexes, ESCRT‐0, ‐I, ‐II and ‐III.^8^ Among them, Hrs from ESCRT‐0 recruits Tsg101 of the ESCRT‐I, and ESCRT‐I is then involved in the recruitment of ESCRT‐III via ESCRT‐II or Alix.^7^ In addition, MVBs and ILVs can form in absence of ESCRT, where tetraspanins such as CD63 and CD81 can be involved.^9^, ^10^, ^11^ MVBs fusing with the plasma membrane release the ILVs, which are then called exosomes.^8^ In this process, Rab27A and Rab27B are critical for the docking of MVBs at the plasma membrane, thereby promoting exosome secretion.^10^

Exosomes, with a size ranging from 50 to 150 nm and 1.13 to 1.19 g/mL of density in sucrose, have a saucer‐shape morphology and contain characteristic proteins such as CD63 and CD9.^12^ Once being engulfed by adjacent and distant cells, exosomes can modify the overall functions of target cells, playing a critical role in autoimmune and chronic inflammatory diseases, such as rheumatoid arthritis and multiple sclerosis.^13^ Recently, the biological capacity of T cell–derived exosomes (T‐exos) in inflammation is increasingly gaining attention. Li et al found that exosomes originated from interleukin (IL)‐12‐stimulated cytotoxic T cells were able to activate bystander CD8^+^ T cells.^14^ Wahlgren et al reported that exosomes released by activated CD3^+^ T cells were capable of promoting proliferation in autologous resting CD3^+^ T cells.^15^ We have previously found that OLP plasma‐derived exosomes enhanced the proliferation and migration of T cells, as well as the secretion of interferon (IFN)‐γ.^16^ However, the effects of human T‐exos on OLP have not been elucidated yet.

In this study, we detected the expression of MVB‐related proteins in OLP T cells, isolated T‐exos and determined their effects on cytokine secretion. We investigated the expression and function of the chemokines which could be up‐regulated by OLP T‐exos and found a chemotactic effect on OLP mononuclear cells. In particular, we analysed the subtype of the migrated T cells and studied the mechanism of this chemotactic effect. Our findings illustrated the role of OLP T‐exos in the inflammation of OLP, which may affect the production of cytokines and aggravate the lesional CD8^+^ T cells infiltration.

## 2 MATERIALS AND METHODS

### 2.1 Participants and sample collection

This study was approved by the Ethical Committee Broad of School and Hospital of Stomatology, Wuhan University (no. 2017028) in accordance with the ethical guidelines of the Declaration of Helsinki. 87 patients clinically diagnosed with OLP by specialists of Oral Medicine and histopathologically diagnosed by oral pathologists, including 37 males and 50 females, were recruited from the Department of Oral Medicine in 2018‐2019. 44 healthy volunteers containing 20 males and 24 females undergoing orthognathic surgery were recruited as controls from the Department of Oral Maxillofacial Surgery in the same period. The average ages of OLP patients and healthy controls (HC) were 48.3 ± 10.3 and 47.2 ± 12.5 years old, respectively. Tissues were taken from the oral lesions of OLP patients or healthy human mucosa of controls. Venous blood was drawn from OLP patients and HCs. All the patients with OLP met the criteria of inclusion and exclusion as we described previously (Appendix 1).^16^ All the HCs were at least 18 years old and signed written informed consent; neither had any systemic disorders nor any other oral lesions; smokers and alcoholics were excluded. The severity of OLP was measured using reticular, atrophic, erosive (RAE) scoring system that we have proposed earlier (Table 1).^17^

**TABLE 1:** RAE scoring system for OLP

Clinical signs	Scoring
Reticular lesions (R)	0 = no white striations
	1 = presence of white striations or keratotic papules
Atrophic areas (A)	0 = no lesion
	1 = lesions less than 1 cm^2^
	2 = lesions from 1 to 3 cm^2^
	3 = lesions greater than 3 cm^2^
Erosive areas (E)	0 = no lesion
	1 = lesions less than 1 cm^2^
	2 = lesions from 1 to 3 cm^2^
	3 = lesions greater than 3 cm^2^
The total score of all 10 areas	Σ*R* + Σ(*A* × 1.5) + Σ (*E* × 2.0)

Note

The oral cavity of each individual was divided into 10 sites: upper/lower labial mucosa, right buccal mucosa, left buccal mucosa, dorsal tongue, ventral tongue, floor of mouth, hard palate mucosa, soft palate/tonsillar pillars, maxillary gingiva, mandibular gingiva.

Abbreviations: OLP, oral lichen planus; RAE, reticular, atrophic, erosive.

### 2.2 Immunohistochemistry

Five µm thick formalin‐fixed, paraffin‐embedded tissue sections were deparaffinized and rehydrated. Antigens were retrieved with sodium citrate buffer using microwave. Sections were treated with hydrogen peroxide and blocked with normal goat serum. After incubating with anti‐MIP‐1α (A7568; ABclonal) and anti‐MIP‐1β (A1671; ABclonal) primary antibodies at 4°C overnight, samples were washed and incubated with HRP polymer conjugated secondary antibody. Immunoreactivity was visualized by diaminobenzidine solution followed by haematoxylin counterstain. The expression level was measured by integrated optical density (IOD) value using Image Pro Plus 6.0 software.

### 2.3 Immunofluorescence

After deparaffinization, rehydration, antigen retrieval and blocking, tissue sections were double‐labelled by anti‐CD63 (A5271; ABclonal)/anti‐Rab27A (ab55667; Abcam)/anti‐Rab27B (13412‐1‐AP; Proteintech) and anti‐CD3 primary antibodies (Proteintech, 60181‐1‐Ig, 17617‐1‐A). Then, the sections were incubated with DyLight 488 (A23210; Abbkine) or Dylight 549 (Abbkine, A23220) and counterstained by 4‐6‐diamidino‐2‐phenylindole (DAPI). The double‐immunofluorescence (IF) stainings were photographed by a fluorescence microscope (Leica).

### 2.4 Quantitative real‐time real‐time polymerase chain reaction

The human peripheral blood mononuclear cells (PBMCs) were isolated by Ficoll‐Paque density gradient centrifugation. T cells were separated from PBMCs using the BD IMag™ Human T Lymphocyte Enrichment Set (557874; BD Biosciences). Total RNA was isolated from tissues or the above‐mentioned T cells using AxyPrep Multisource Total RNA Miniprep kit (Axygen). cDNA was synthesized using the PrimeScript^TM^ RT reagent Kit with gDNA Eraser (RR047A; TAKARA), and 16 ng of cDNA was loaded for each PCR reaction. Primers were listed in Table 2. The quantitative real‐time polymerase chain reaction (qRT‐PCR) was performed using the TB Green Premix Ex Taq^TM^ (RR420A; TAKARA) detection assay on an CFX Connect Real‐Time PCR Detection System (Bio‐Rad). Relative gene expression was normalized to the expression of GAPDH using the 2^−ΔΔCt^ method.

**TABLE 2:** Primers designed for qRT‐PCR

	Forward	Reverse
CD63	5′‐GAAAATCCCTTCCATGTCGAAG‐3′	5′‐ATTCCCAAAACCTCGACAAAAG‐3′
CD81	5′‐GCCAAGGATGTGAAGCAGTT‐3′	5′‐TAAGCGTCTCGTGGAAGGTC‐3′
Rab27A	5′‐GAAAGAGGAGGAAGCCATAGCAC‐3′	5′‐CATGACCATTTGATCGCACCAC‐3′
Rab27B	5′‐CCAGATCAGAGGGAAGTCAATG‐3′	5′‐CCAGTTGCTGCACTTGTTTC‐3′
Tsg101	5′‐GAGAGCCAGCTCAAGAAAATGG‐3′	5′‐TGAGGTTCATTAGTTCCCTGGA‐3′
Alix	5′‐CTGGAAGGATGCTTTCGATAAAGG‐3′	5′‐AGGCTGCACAATTGAACAACAC‐3′
IFN‐γ	5′‐ATGTCCAACGCAAAGCAATAC‐3′	5′‐ACCTCGAAACAGCATCTGAC‐3′
GAPDH	5′‐CTTTGGTATCGTGGAAGGACTC‐3′	5′‐CAGTAGAGGCAGGGATGATGTT‐3′

Abbreviation: qRT‐PCR, quantitative real‐time polymerase chain reaction.

### 2.5 Cell culture and treatment

T cells were maintained in T cells Serum‐free Medium (RC‐003‐500; STEMERY) after separation, supplemented with 20 ng/mL rh‐IL‐2 (589102; Biolegend), 100 U/mL penicillin and 100 μg/mL streptomycin and activated with 5 μg/mL plate‐coated CD3 antibody (16‐0037‐85; eBioscience) and 2 μg/mL free CD28 antibody (16‐0037‐28; eBioscience). For chemotaxis assays, PBMCs were cultured in the same condition as T cells and activated for 3 days. Jurkat cells were incubated in RPMI 1640 supplemented with 10% exosome‐free foetal calf serum and activated with CD3 and CD28 for 48 hours.

### 2.6 Purification of exosomes

Supernatants of human T cells were collected from 5‐6 days cell cultures, and exosomes were purified as followed. Briefly, culture supernatants were centrifuged at 2000 *g* for 35 minutes to remove cell debris and dead cells. Supernatants were collected and filtered through 0.22 μm filters and then centrifuged at 100 000 *g* for 70 minutes at 4°C (Optima XPN‐100; Beckman Coulter). The pelleted exosomes were suspended in PBS and then ultracentrifuged at 100 000× g for another 70 minutes.

### 2.7 Characterization of purified exosomes

Purified exosomes suspended in PBS were dropped on a copper mesh and incubated at room temperature for 5 minutes. After fixing with 2% uranyl acetate for 3 minutes, samples were visualized using a transmission electron microscope (TEM; Tecnai G2 Spirit BioTwin, 80 kV; FEI). The size of exosomes was identified using nanoparticle tracing assay (ZetaVIEW S/N 17‐310; PARTICLE METRIX) and analysed by ZetaVIEW 8.04.02 software. Western blot was performed to detect the exosomal marker CD63 (CBL553; Millipore) and CD9 (555370; BD Science).

### 2.8 Confocal microscopy

Purified T‐exos were labelled with PKH67 Fluorescent Cell Linker Kits (MIDI67; Sigma‐Aldrich) and then diluted with PBS and ultracentrifuged at 120 000 *g* for 70 minutes at 4°C to remove unbound dye. After staining with 10 μmol/L Dil fluorescent cell membrane probe (C1036; Beyotime) for 30 minutes and washing with PBS, 5 × 10^5^/mL Jurkat cells were co‐cultured with PKH‐67 labelled exosomes in confocal dishes. At the end of incubation, cells were washed for three times, fixed with 4% paraformaldehyde for 15 minutes, stained with DAPI and observed under a confocal laser scanning microscope (Olympus Optical Co Ltd.) or measured by FACS Calibur flow cytometry.

### 2.9 Luminex xMAP‐Based assay

Activated Jurkat cells cultured at 5 × 10^5^ cells per well were treated with 50 μg T‐exos for 48 hours. After that, supernatants were collected and analysed using Bio‐Plex MAGPIX System (#M500KCAF0Y; Bio‐Rad) according to the manufacturer's protocol. Specifically, 50 μL supernatants of Jurkat cells were loaded and tested. The cytokine concentrations were calculated using Bio‐Plex Manager software 6.1 (Bio‐Rad Laboratories, Inc)

### 2.10 Enzyme‐linked immunosorbent assay

The expression level of MIP‐1α and MIP‐1β in human plasma was measured using ELISA Kits (E‐EL‐H0021c, E‐EL‐H0022c; Elabscience) according to manufacturer's instructions.

### 2.11 Flow cytometry

Whole blood was stained with FITC‐labelled CD3 (300306; Biolegend), APC‐labelled CD4 (357408; Biolegend), APC/CY7‐labelled CD8 (344714; Biolegend), PE/CY7‐labelled CCR1 (362914; Biolegend), PE‐labelled CCR3 (310706; Biolegend) and PE‐labelled CCR5 (359106; Biolegend) antibodies for 15 minutes at room tempareture in dark, followed by red blood cells lysate for another 15 minutes using lysing buffer (555899; BD Biosciences). Cells were fixed by 4% polyoxymethylene and measured via flow cytometry. Results were analysed by Flowjo V10 software.

### 2.12 Chemotaxis assay

Chemotaxis assays were performed using a 24‐well transwell chamber with 5‐μm pores (3421; Corning). Peripheral blood mononuclear cells were resuspended in T cells Serum‐free Medium (10^6^ cells/mL). CCR1 inhibitor BX‐471 (T2375; TargetMol) and CCR5 inhibitor maraviroc (TargetMol, T6016) were dissolved in dimethyl sulphoxide (DMSO). 100 μL T‐cell suspension was added to the upper chambers with or without 20 μmol/L BX‐471 or maraviroc. 500 μL medium was added to the lower chambers with or without 200 ng rh‐MIP‐1α (C061; Novoprotein) or rh‐MIP‐1β (300‐09‐50; Pepro Tech). The chemotaxis plates were then incubated at 37°C for 12 hours. After incubation, the upper chamber was stained with crystal violet, photographed and analysed by Image Pro Plus 6.0, and the cells in the lower chambers were collected and tested by flow cytometry.

### 2.13 Statistical analysis

All data were analysed by Graphpad Prism 7.0. When data were normally distributed and showed homogeneity of variance, significance of mean differences was determined by unpaired Student's *t* test (two groups) or one‐way ANOVA with Tukey's multiple comparison test (more than two groups); otherwise, they were calculated by non‐parametric Mann‐Whitney *U* tests (two groups) or Kruskal‐Wallis test (more than two groups). Spearman's correlation test was used to examine the clinical correlations. Experimental data were presented as mean ± SEM. Differences were considered statistically significant at *P* < 0.05.

## 3 RESULTS

### 3.1 MVB‐related genes and proteins were significantly up‐regulated in OLP T cells and correlated with disease severity

**FIGURE 1:**
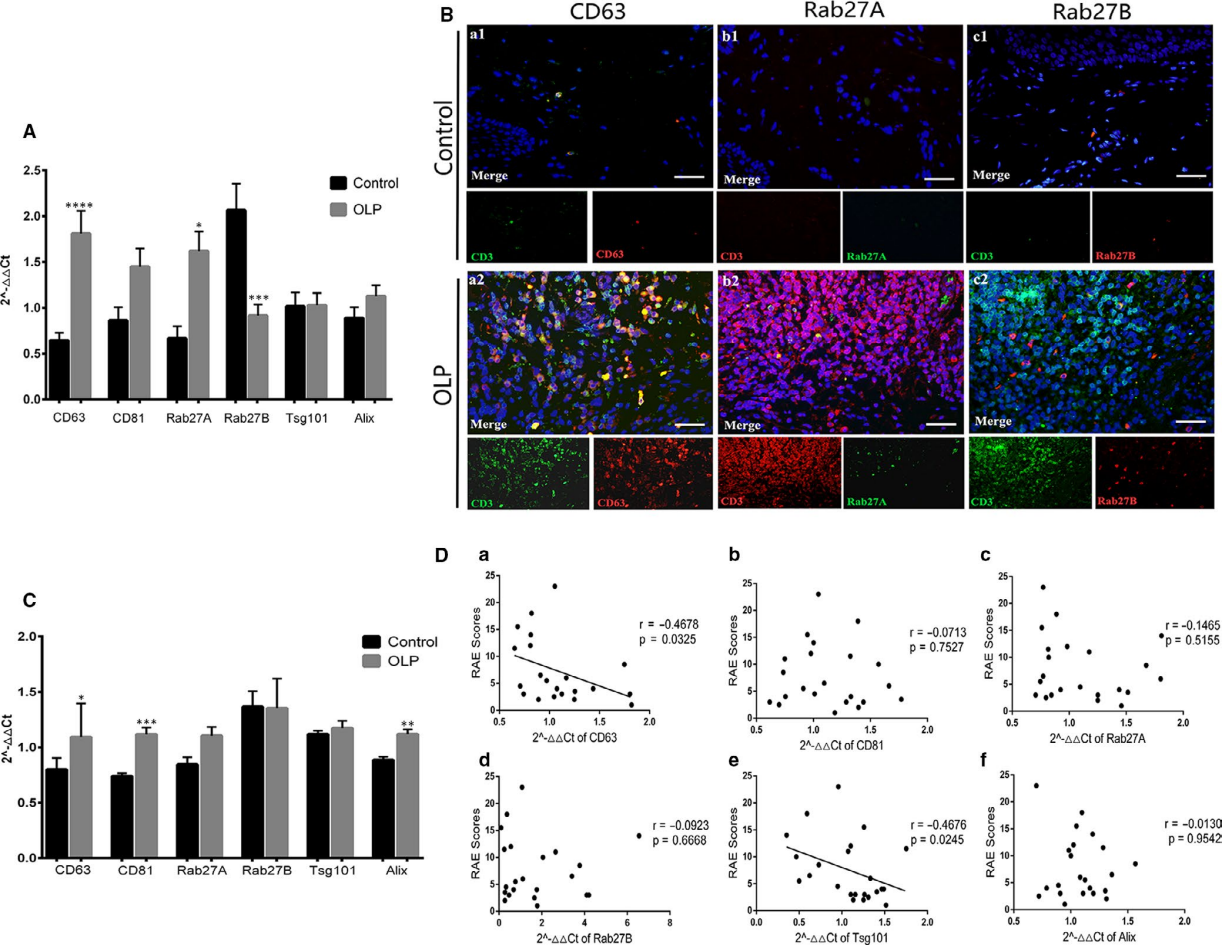
Expression of CD63, CD81, Rab27A, Rab27B, Alix and Tsg101 in oral lichen planus (OLP) T cells. A, Gene expressions of CD63, CD81, Rab27A, Rab27B, Alix and Tsg101 in OLP (n = 34) and control (n = 14) tissues were measured by qRT‐PCR. B, Co‐localization of CD63 (red)/CD3 (green), Rab27A (green)/CD3 (red) and Rab27B (red)/CD3 (green) in OLP (n = 14) and control tissues (n = 9) was detected by IF. Nuclei were stained with DAPI (blue). Scale bar, 25 µm. Magnification, 40×. C, Gene expressions of CD63, CD81, Rab27A, Rab27B, Alix and Tsg101 in OLP (n = 22) and control (n = 10) peripheral T cells were analysed using qRT‐PCR. D, Correlation between the gene expression of CD63, CD81, Rab27A, Rab27B, Alix and Tsg101 in OLP peripheral T cells (n = 22) and the RAE scores. *, vs control group. *P < 0.05, **P < 0.01, ***P < 0.001, ****P < 0.0001. qRT‐PCR, quantitative real‐time polymerase chain reaction. IF, immunofluorescence

To explore if there was a connection between exosomes and T cells, we firstly made a preliminary analysis of 6 representative MVB‐related genes by qRT‐PCR: CD63, CD81, Rab27A, Rab27B, Alix and Tsg101. In comparison with HCs, the gene expression of CD63 and Rab27A was significantly up‐regulated in OLP, while Rab27B gene expression was significantly down‐regulated (Figure 1A). In OLP lesions, CD63 was densely infiltrated in the lamina propria and markedly co‐localized with T cells (Figure 1B). Rab27B expression was increased and diffusely distributed in the lamina propria of OLP, while Rab27A was rarely seen in both OLP and control groups, and few of Rab27A/B was co‐localization with T cells (Figure 1B). The expression of the above‐mentioned 6 genes in peripheral T cells was also analysed. The gene level of CD63, CD81 and Alix in OLP T cells was significantly higher than that in controls (Figure 1C). Clinical correlation analysis showed that, in OLP lesions, the mRNA level of CD63 was positively correlated with the corresponding RAE scores, while that of Rab27B was in a negative correlation with the disease severity (Appendix 2). On the contrary, the gene level of CD63 in peripheral T cells was negatively correlated with the RAE scores (Figure 1D‐a), so was the gene expression of Tsg101 (Figure 1D‐e). These data suggested that exosomes released by OLP T cells may be implicated in the pathogenesis of OLP.

### 3.2 Identification and internalization of exosomes derived from human peripheral T cells

**FIGURE 2:**
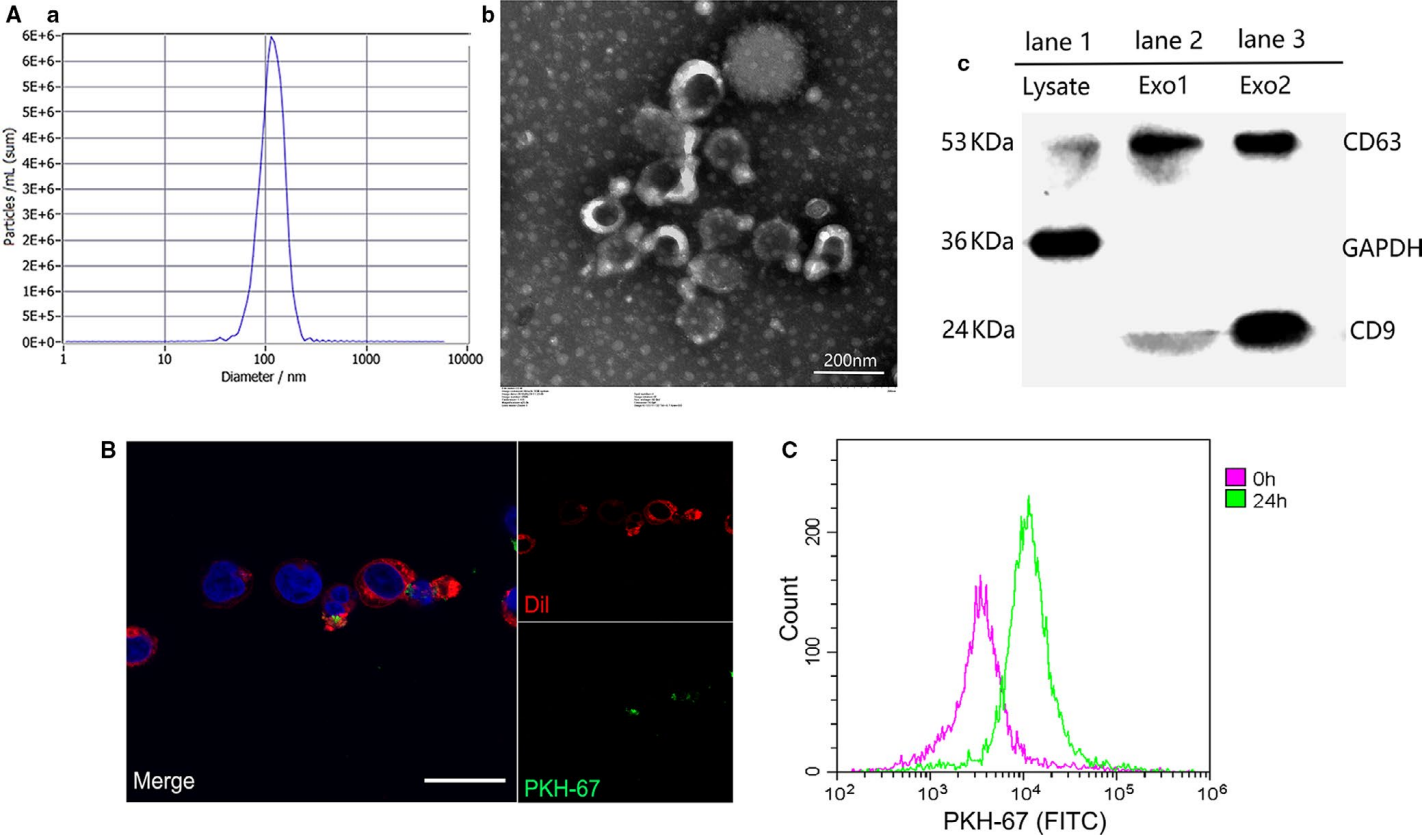
Identification and internalization of T cell–derived exosomes. A, The size of the extracellular vesicles was tested by nanoparticle tracing assay (a, b), the morphology of exosomes was observed by transmission electron microscopy (c), and the expression of surface markers CD63 and CD9 was detected using Western blot assay (d) where 25 μg lysate of T cells (lane 1) or T cell–derived extracellular vesicles (lane 2 and 3) were loaded. B, After co‐culture for 24 h, the internalization of PKH‐67 labelled exosomes (green) by Dil labelled Jurkat cells (red) was observed by confocal microscope. Scale bar, 30 μm. C, Jurkat cells that engulfed T‐exos (PKH‐67 positive) after co‐incubation were detected by flow cytometry. Each experiment was repeated three times

We then isolated T‐exos by ultracentrifugation and identified it on the basis of morphology, size distribution and membrane composition. Nanoparticle tracing assay showed that the size of the T cell–derived extracellular vesicles was homogeneously distributed with an average size of 118.7 ± 32.1 nm and a diameter peak at 117 nm. A classic saucer‐shape morphology of these vesicles was observed under the TEM. Besides, the amount of exosomal markers CD63 and CD9 was significantly higher in exosomes than in the lysate of T cells (Figure 2A).

To determine whether T‐exos could be internalized, PKH‐67 labelled T‐exos (green) were incubated with Dil stained Jurkat cells (red). As the results showed, T‐exos were incorporated by Jurkat cells after incubating for 24 hours (Figure 2B,C).

### 3.3 OLP T‐exos promoted the secretion of MIP‐1α/β

**FIGURE 3:**
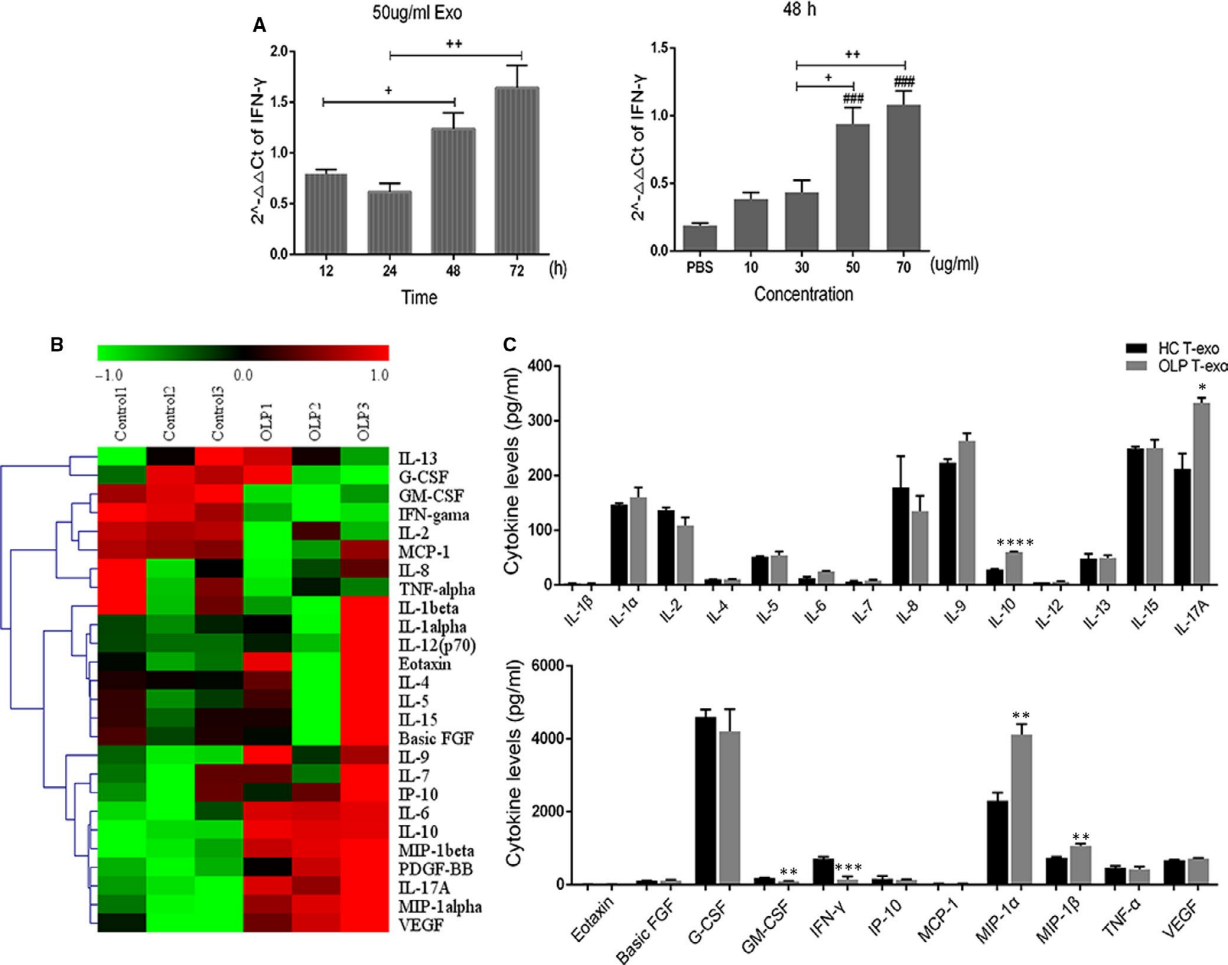
Oral lichen planus (OLP) T cell–derived exosomes regulated the differentiation and cytokines secretion of Jurkat cells. A, 50 μg/mL HC T‐exos (n = 3) were co‐cultured with Jurkat cells for different times, or different concentrations of HC T‐exos (n = 3) were incubated with Jurkat cells for 48 h. After that, the mRNA of IFN‐γ in Jurkat cells was measured by qRT‐PCR. B, After incubation with 50 μg/mL HC T‐exos (n = 3) or OLP T‐exos (n = 3) for 48 h, the expression of 26 cytokines in the supernatants of the co‐culture systems were detected using luminex assay. The heat map illustrates relative cytokine release. C, The expression of the above‐mentioned 26 cytokines was analysed using SPSS 17.0 software. ^+^, difference between the two groups; ^#^, vs PBS group; *, vs control group. ^+^ *P*, ^#^ *P*, *P < 0.05; ^++^ *P*, ^##^ *P*, **P < 0.01; ^###^ *P*, ***P < 0.001; ****P < 0.0001. qRT‐PCR, quantitative real‐time polymerase chain reaction

To investigate the functions of T‐exos on the pathogenesis of OLP, we co‐cultivated T‐exos with Jurkat cells and evaluated the cytokines secreted by the co‐cultured cells. After incubation with HC T‐exos, the gene level of IFN‐γ in Jurkat cells was significantly up‐regulated in a dose‐ and time‐dependent manner. The gene expression of IFN‐γ in Jurkat cells incubated with 50 μg/mL HC T‐exos for 48 or 72 hours, or in Jurkat cells cultured with 50 or 70 μg/mL HC T‐exos for 48 hours was significantly higher (Figure 3A).

We therefore incubated Jurkat cells with 50 μg/mL T‐exos for 48 hours, and detected the expression pattern of 26 cytokines associated with the inflammation of OLP in the supernatants of the co‐culture systems. The relative cytokines expression in the supernatants was different between the OLP T‐exos and HC T‐exos treated groups. To be specific, the level of granulocyte‐macrophage colony‐stimulating factor (GM‐CSF) and IFN‐γ was relatively lower while the IL‐6, IL‐10, IL‐17A, MIP‐1α/β and vascular endothelial growth factor was relatively higher in the supernatants of OLP T‐exos treated Jurkat cells than that of HC T‐exos treated group (Figure 3B). Statistical analysis showed that in the supernatants of Jurkat cells treated by OLP T‐exos, the up‐regulation of IL‐10, IL‐17A, MIP‐1α and MIP‐1β and the down‐regulation of GM‐CSF and IFN‐γ, were of statistical significance (Figure 3C). Among these aberrantly expressed cytokines, MIP‐1α and MIP‐1β which are CC chemokines changed most obviously, indicating that OLP T‐exos may contribute to the migration of T cells by increasing the secretion of MIP‐1α/β.

### 3.4 Increased expression of MIP‐1α/β and CCR1/5^+^ T cells in OLP

**FIGURE 4:**
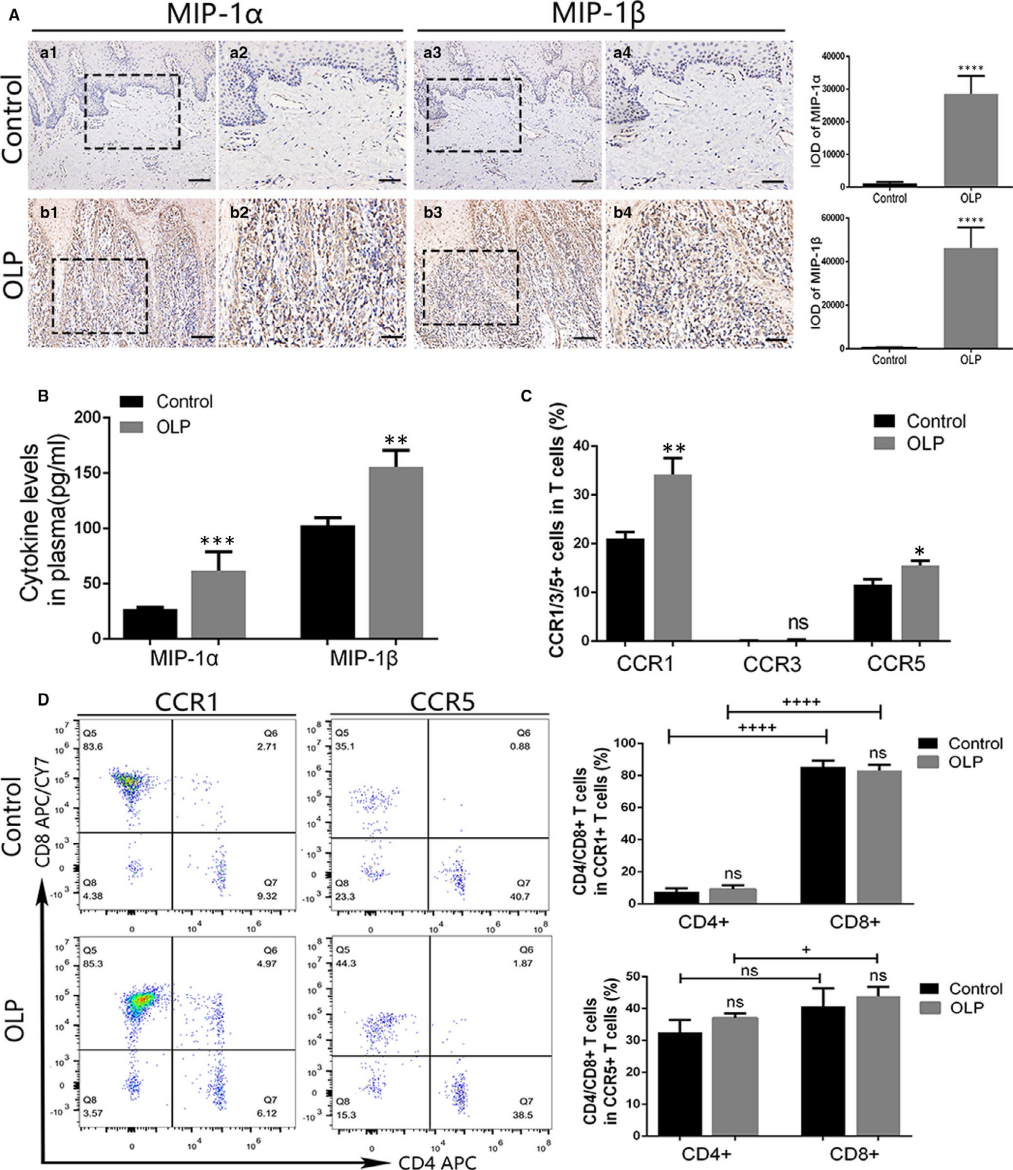
The expression of macrophage inflammatory protein (MIP)‐1α/β in oral lichen planus (OLP) and the expression of CCR1/3/5 in OLP peripheral T cells. A, The expression of MIP‐1α/β in OLP (n = 14) and control (n = 9) mucosal tissues was identified by IHC and assessed by IOD value. Magnification: a1, a3, b1, b3, 20×, scale bar, 50 μm; a2, a4, b2, b4, 40×, scale bar, 25 μm. B, Cytokine levels of MIP‐1α and MIP‐1β in the plasma of OLP (n = 12) and control (n = 8) were detected by ELISA. C, Membrane expression of CCR1/3/5 on OLP (n = 10) and control (n = 8) peripheral T cells was measured using flow cytometry. D, The proportion of CD4^+^ and CD8^+^ T cells in CCR1^+^ or CCR5^+^ T cells was analysed via flow cytometry. ^+^, difference between the two groups; *, vs control group. ^+^ *P*, *P < 0.05; **P < 0.01; ***P < 0.001; ^++++^ *P* < 0.001. ELISA, enzyme‐linked immunosorbent assay; IHC, immunohistochemistry; IOD, integrated optical density; ns, no significant difference

To determine the effects of MIP‐1α/β on OLP, we first detected the expression of MIP‐1α/β and their receptors CCR1/3/5. The expression of MIP‐1α and MIP‐1β was remarkably increased in the OLP lamina propria and plasma compared with HCs (Figure 4A,B). In peripheral T cells, CCR1 and CCR5 were highly expressed on OLP T cells when compared with controls, whereas the expression of CCR3 showed no significance between the two groups and expressed on only 0%‐0.8% T cells (Figure 4C; Appendix 3). We further analysed the proportion of CD4^+^ and CD8^+^ cells in CCR1/5^+^ T cells and found that the majority of CCR1^+^ T cells were CD8^+^ T cells, and in OLP the percentage of CCR5^+^CD8^+^ T cells was significantly higher than that of CCR5^+^CD4^+^ T cells (Figure 4D).

### 3.5 MIP‐1α/β induced the migration of OLP CD8^+^ T cells via CCR1 and CCR5

**FIGURE 5:**
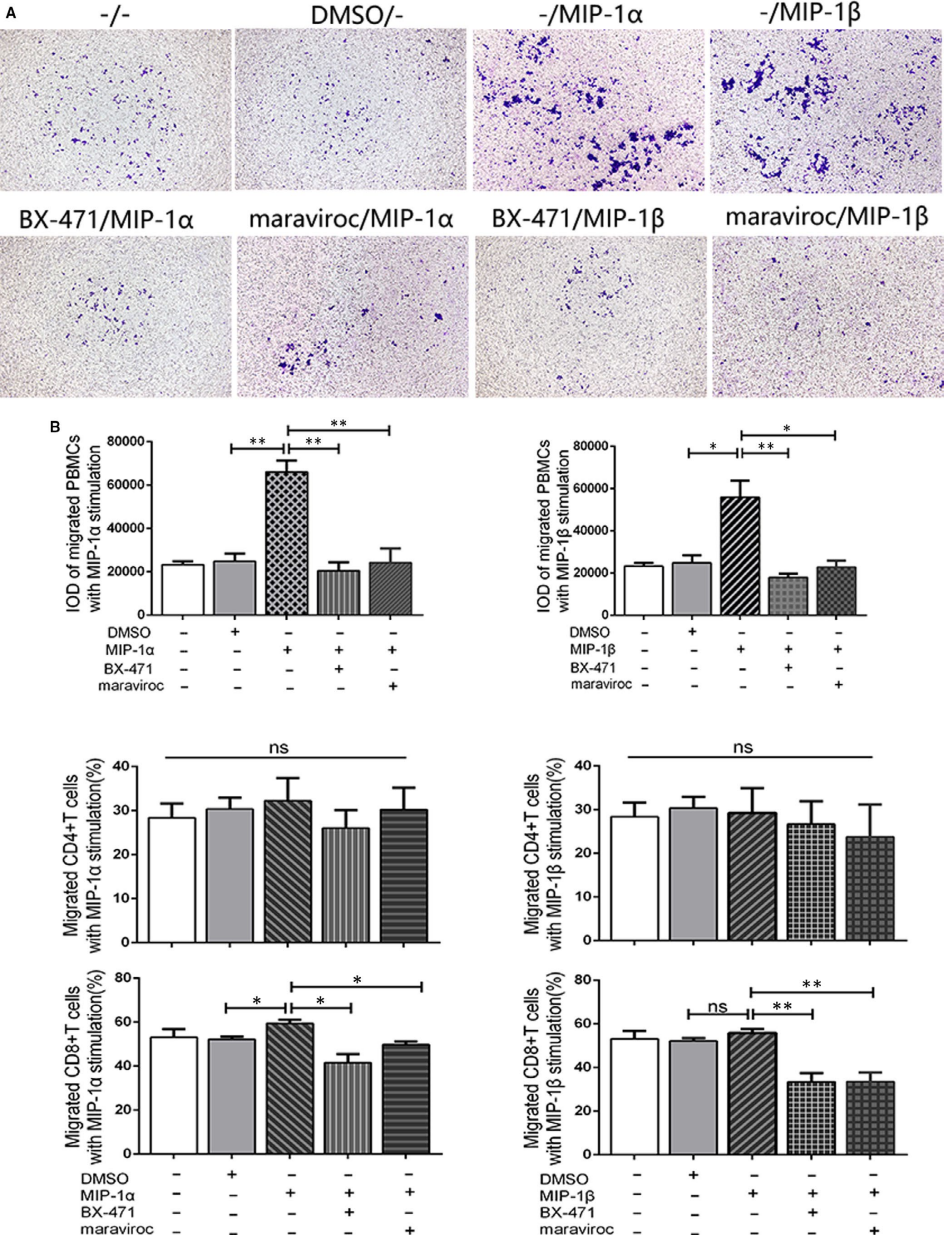
Macrophage inflammatory protein (MIP)‐1α/β induced the migration of oral lichen planus (OLP) CD8^+^ T cells via CCR1 and CCR5. A, PBMCs (n = 4) passing through the pores were stained with crystal violet and assessed by IOD value. Magnification, 20×. B, The proportion of MIP‐1α/β recruited CD4^+^ and CD8^+^ T cells in the lower chambers was measured by flow cytometry. Data were presented as mean ± SEM. *, difference between the two groups. *P < 0.05, **P < 0.01. BX‐471, CCR1 inhibitor; maraviroc, CCR5 inhibitor; IOD, integrated optical density; ns, no significant difference

Then, we performed transwell assay and flow cytometry to test the chemotactic effect of MIP‐1α/β on OLP T cells and its possible mechanism. The results showed that MIP‐1α/β stimulation significantly enhanced the migration of OLP PBMCs, and the inhibition on CCR1/5 markedly decreased the total number of migrated PBMCs (Figure 5A). Although MIP‐1α treatments elevated the proportion of migrated CD8^+^ T cells in comparison with the DMSO groups, the alteration was slight (Figure 5B; Appendix 4). Before inhibiting CCR1/5, the predominant subtype in the lower chambers was CD8^+^ T cells. Inhibition on the CCR1 or CCR5 significantly diminished the proportion of migrated CD8^+^ T cells; however, it did not alter the percentage of CD4^+^ T cells in the lower chambers (Figure 5B).

## 4 DISCUSSION

Upon TCR activation, the production of exosomes from T cells would be highly increased,^18^ which subsequently could be internalized by in situ or remote cells and display immunologic functions.^19^ Wang et al found that exosomes derived from exhausted CD8^+^ T cells were able to damage the proliferation and cytokine production such as IFN‐γ and IL‐2 of normal CD8^+^ T cells, impairing the anticancer function of normal CD8^+^ T cells.^20^ In type I diabetes, T‐exos could lead to the death pancreatic β cells and increase the expression of chemokine genes, which may promote the autoimmune attack.^21^ Here, we first identified the potential linkage between exosomes and OLP T cells by detecting the aberrant expression of MVB‐related proteins in OLP T cells. Then, we purified T‐exos and demonstrated that OLP T‐exos could inhibit the secretion of GM‐CSF and IFN‐γ but promote the production of IL‐10 and IL‐17A by Jurkat cells. Especially, we found a significant contributory effect of OLP T‐exos on the release of MIP‐1α and MIP‐1β, which further elicited the migration of OLP CD8^+^ T cells through CCR1 and CCR5.

A dense, band‐like infiltration of lymphocytes is the most distinct histopathological characteristic of OLP.^22^ CD4^+^ T cells are the dominated lymphocytes in the subepithelium and lamina propria, while in the intra‐epithelium of OLP lesions, the majority of lymphocytes infiltrated are activated CD8^+^ lymphocytes.^3^, ^23^ The mechanism by which CD8^+^ T cells migrated to the lesional sites, however, has not been completely clarified.^24^ MIP‐1α and MIP‐1β, members of the CC chemokine subfamily, could be produced by lymphocytes, macrophages and other immune cells, exerting chemotactic effect on T cell via binding to its cell surface receptors CCR1, CCR3 and CCR5.^25^ Castellino et al revealed that in vivo CD4^+^ T cells produced MIP‐1α and MIP‐1β upon activation and naive CD8^+^ T cells up‐regulated CCR5 after immunization; as a result, cognate MIP‐1α/β dramatically contributed to the accumulation of CD8^+^ T cells to the sites of dendritic cell‐CD4^+^ T cell interaction via CCR5.^26^ Here, we demonstrated the promotional effects of OLP T‐exos on MIP‐1α/β secretion, the marked expression of MIP‐1α/β in OLP and that of CCR1/5 on CD8^+^ T cells, suggesting the close relation among T‐exos, MIP‐1α/β and CD8^+^ T cells. MIP‐1α/β stimulation promoted the migration of OLP PBMCs; however, it did not obviously alter the proportion of migrated CD4^+^ and CD8^+^ T cells, indicating that MIP‐1α/β increased the migration of overall T cells rather than changed the percentage of certain subtype. The mechanisms involved in the generation of MIP‐1α/β by T cells are believed to be related with the toll‐like receptor (TLR) 2 and/or TLR4,^27^, ^28^ which initiated the activation of nuclear factor‐kappa B (NF‐κB) signalling pathways.^27^, ^29^, ^30^ It is noteworthy that in OLP the expression of TLR2 was enhanced in lesional tissues and PBMCs,^31^ and we have found a strong nuclear expression of p65 (a subunit of NF‐kB) which symbolize the activation of NF‐κB in lesional infiltrated lymphocytes.^32^ Thus, it is possible that OLP T‐exos target the TLR2 on peripheral or local T cells and thereby induce the production of MIP‐1α/β through NF‐κB pathway, ultimately driving the trafficking of CD8^+^ T cells via CCR1/5.

In addition to the enhanced expression of MIP‐1α/β, we found an elevation of IL‐10 and IL‐17A and a reduction of GM‐CSF and IFN‐γ in the supernatants of OLP T‐exos treated Jurkat cells. The aberrant expression and potential functions of these cytokines in OLP have been verified by multiple researches. IFN‐γ may activate CD8^+^ T cells and maintain the differentiation and proliferation of Th1 cells.^3^ IL‐10, on the contrary, is an anti‐inflammatory Th2 cytokine with inhibitory effect on the production of IL‐2 and IFN‐γ.^33^ Both IFN‐γ and IL‐10 were found up‐regulated in OLP lesions and down‐regulated in OLP PBMCs.^34^ Researchers also have identified the up‐regulation of IL‐17A in OLP lesions and saliva,^34^, ^35^, ^36^ and we have previously found that exogenous IL‐17 could significantly enhance the mRNA expressions of CCL‐20, IL‐8 and tumour necrosis factor‐α in normal oral keratinocytes.^37^ To date, there has not study regarding GM‐CSF in OLP. As a multifunctional cytokine with pro‐inflammatory functions,^38^ GM‐CSF is worth investigating in OLP in the future. The present study suggested that OLP T‐exos may regulate the inflammatory status of OLP by increasing the secretion of IL‐10 and IL‐17A and decreasing the production of GM‐CSF and IFN‐γ.

Conclusively, our study demonstrated that OLP T‐exos affected the cytokine secretion of T cells by down‐regulating GM‐CSF and IFN‐γ and up‐regulating IL‐10, IL‐17A and MIP‐1α/β. Notably, MIP‐1α and MIP‐1β elevated by OLP T‐exos may in turn attract mononuclear cells and recruit CD8^+^ T lymphocytes via CCR1 and CCR5, indicating that OLP T‐exos might contribute to the development of OLP by increasing the infiltration of T lymphocytes in lesional sites.

## CONFLICT OF INTEREST

The authors state no conflict of interest.

## AUTHOR CONTRIBUTIONS

**Jing‐ya Yang:** Conceptualization (lead); Data curation (lead); Formal analysis (lead); Investigation (lead); Methodology (lead); Resources (lead); Software (lead); Validation (lead); Visualization (lead); Writing‐original draft (lead); Writing‐review & editing (lead). **Jing Zhang:** Conceptualization (supporting); Data curation (supporting); Investigation (supporting); Methodology (supporting); Resources (supporting); Supervision (supporting); Writing‐review & editing (supporting). **Rui Lu:** Conceptualization (supporting); Data curation (supporting); Investigation (supporting); Methodology (supporting); Resources (supporting); Software (supporting); Validation (supporting). **Ya‐qin Tan:** Conceptualization (supporting); Investigation (supporting); Methodology (supporting); Resources (supporting); Software (supporting); Validation (supporting); Writing‐review & editing (supporting). **Ge‐fei Du:** Data curation (supporting); Formal analysis (supporting); Methodology (supporting); Resources (supporting); Supervision (supporting); Validation (supporting). **Gang Zhou:** Conceptualization (supporting); Data curation (supporting); Funding acquisition (lead); Investigation (supporting); Project administration (lead); Resources (supporting); Supervision (supporting); Writing‐review & editing (supporting).

## Data Availability

Data are available on request from the authors. The data that support the findings of this study are available from the corresponding author upon reasonable request.

## APPENDIX 1 The inclusion and exclusion criteria for oral lichen planus (OLP) patients

**Table:**

The inclusion criteria:
1. At least 18 years of age and signed written informed consent
2. Clinically and histopathologically diagnosed with OLP according to the modified WHO diagnostic criteria^36^ and the newly proposed diagnostic process^37^
The exclusion criteria
1. A history of smoking or alcohol abuse
2. Pregnancy, lactation
3. Patient with infectious, allergic, cardiovascular, hematological, endocrine, metabolic and immune‐related diseases
4. Exposure to systemic or topical anti‐inflammatory, immunomodulatory drugs at least within 3 months
5. Patient with concomitant other oral lesions
6. Participant with oral lichenoid reactions, including lichenoid contact reactions, lichenoid drug eruptions and lichenoid reactions of graft‐vs‐host disease
7. Presence of epithelial dysplasia in histopathological examination

## APPENDIX 2

Correlation between the gene expression of CD63, CD81, Rab27A, Rab27B, Alix and Tsg101 in oral lichen planus peripheral lesions (n = 34) and the reticular, atrophic, erosive scores.

## APPENDIX 3

Gating strategy. Representative contour plots showing the general gating strategy used to identify the CCR1^+^/CCR3^+^/CCR5^+^ T cells from human peripheral blood.

## APPENDIX 4

Gating strategy. Representative contour plots showing the general gating strategy used to analyse the proportion of the CD4^+^/CD8^+^ T cells in the lower transwell chamber.
